# Data-driven modeling of spring discharge in a dynamic karst aquifer using multivariate models and spectral analysis

**DOI:** 10.1371/journal.pone.0351623

**Published:** 2026-08-11

**Authors:** Gianna Vivaldo, Brunella Raco, Matia Menichini, Giulio Masetti, Luca Fibbi, Bernardo Gozzini, Daniele Grifoni, Marco Doveri

**Affiliations:** 1 Institute of Geosciences, National Research Council, Pisa, Italy; 2 National Biodiversity Future Centre, Palermo, Italy; 3 Institute of Bioeconomy, National Research Council, Sesto Fiorentino (FI), Italy; 4 LaMMA Consortium, Sesto Fiorentino (FI), Italy; 5 Department of Earth Sciences, University of Pisa, Pisa, Italy; University of Nevada Las Vegas, UNITED STATES OF AMERICA

## Abstract

Karst aquifers are highly sensitive to climate change due to their complex internal structure, which makes them both reactive and difficult to model. This study proposes a transdisciplinary decision-support analytical framework to support long-term groundwater management in a dynamic karst system in northwestern Tuscany (Italy). The approach combines time- and frequency-domains techniques – multivariate regression models and singular spectrum analysis, respectively – to characterize both short-term system memory and the low-frequency variability of the spring discharge. The methodology was applied to the Cartaro spring (Apuan Alps) using a 18-year dataset of discharge and meteorological variables (precipitation and temperature). Time-domain analysis showed that meteorological variables alone cannot fully explain the long-term variability of spring discharge, and that accounting for the short memory of the karst system (about five days) significantly improved the performance of the classical models. Furthermore, spectral decomposition allowed us to extract the significant annual and semi-annual oscillatory components with modulated amplitude, highlighting the role of low-frequency climatic variability in controlling spring behavior. By combining the results from the time and frequency domains, we fine‑tuned a forecasting procedure that enables statistically reliable annual predictions of long‑term average discharge trends, while limiting noise propagation and without relying on physical governing equations. The research is transdisciplinary, as it was developed from its preliminary phases in collaboration with stakeholders and local managers. The methodological workflow can be applied to other karst aquifers, provided that it is recalibrated using the site-specific hydro-climatic and discharge data.

## Introduction

Climate change is strongly affecting water resources supply due to the intensification of the hydrological cycle, which in turn leads to hydroclimatic stresses expected to increase in the next years in several regions [1] because of climate extremes, such as droughts and floods, tied to global warming [2–6]. Water supply scarcity and surface water contamination are among the most impacting consequences of climatic extremes from the socio-economical point of view, bringing to a difficult management of superficial water resources. In the last fifty years, because of the drawbacks affecting superficial waters, the exploitation of groundwater resources has been highly intensified, pushed by the growing demographic pressure [7]. This brought to a degradation of water quality, besides groundwater systems being objectively more resilient to climate change with respect to surface waters [8].

In a growing number of countries, the safeguarding of drinking water supplies is strictly linked with sustainable usage of groundwater resources, which in turn are the main source for water supply, since most terrestrial water resources are stored underground [9–12]. Worldwide, more than two billion people, in fact, depend on groundwater resources for daily water supply [13]. In the European Union (EU), around 70% of the water used for drinking water is stored underground. [14]. Groundwater withdrawals for irrigation are also significant and increasing [15]. Nearly 75% of groundwater comes from carbonate aquifers, which are particularly vulnerable to global warming due to their karst features that make them particularly sensitive and reactive to external stimuli. Karst aquifers host important water resources, which feed large springs captured by civil aqueducts, thus playing a crucial role in water supply in many countries [16–17].

Traditional modeling approaches often struggle to capture the non-linear, multi-scale dynamics of these systems, especially in the absence of detailed subsurface data. Despite their importance, groundwater systems (as opposed to surface water) remain underrepresented in climate-related hydrological studies. Several theoretical approaches have been proposed to define sustainable groundwater yield [18–20]. However, only a few experimental and practical applications have been explored in the framework of fractured aquifers. Over the last decades, several conceptual and numerical approaches have been developed to characterize the behavior of karstic aquifers [21–32], but their applicability is limited by the intrinsic heterogeneity, anisotropy and non-linear dynamics evolution of karst environments [29,32].

Among data-driven approaches, multiple linear regression models represent a widely used and transparent framework to investigate the relationship between hydro-meteorological variables and spring discharge in the time-domain (refer to [33] and reference therein), also in karst springs [34]. Their relatively simple structure allows the contribution of individual predictors to be interpreted, which is particularly useful in environmental management contexts. In addition, the inclusion of autoregressive terms, using both traditional and advanced models, makes it possible to account for persistence and memory effects typical of hydrological time series, which are particularly relevant in karst aquifers where discharge reflects previous hydrological states of the system [34,35].

To further address this issue, various approaches have been proposed over the years. Lumped-parameter models have been introduced to simulate the overall hydrological response of karst systems, especially when few data are available and simplified representations are needed [36,37]. These models do not require detailed knowledge of the subsurface geometry. However, they are less effective for long-term predictions under changing boundary conditions, such as those determined by climate change as in our case study.

In recent decades, machine learning has been widely applied to hydrological studies proving to be a good alternative to the classical modeling approach [38–40]. Artificial neural networks (ANNs) have proven effective in various hydrological applications, including forecasting surface flows and floods, watershed management, rainfall-runoff modeling, water quality assessment, and unsaturated zone flow simulation [41–49]. The combination of fuzzy logic and neural networks have been proposed to offer new perspectives for modeling daily discharge responses in aquifer systems [39,50]. Karst aquifer discharge mainly occurs at conduit-flow springs, which are highly variable in space and time. These springs reflect an integrated response to water movement through diverse flow paths and mixing processes across the karst system at multiple temporal scales [30]. As a result, they exhibit a well-known memory effect involving all underlying hydrological processes [21,51], which are influenced by recharge and discharge dynamics, and by both short- and long-term climatic and meteorological variations.

In the frequency-domain, classical spectral methods – mainly cross-correlation analysis or Fast Fourier Transform-derived methodologies – have been used to study precipitation – discharge relationships in karst systems [51–54]. These analyses were able to reveal the memory effect in rainfall input, influenced by the aquifer's degree of karstification and internal storage dynamics. Nevertheless, classical spectral methods present several limitations. First, they are parametric approaches which require fitting the experimental data with an assumed *a-priori* model. Second, they are unable to represent amplitude and frequency modulation of the dominant oscillatory components in a real signal. In addition, the choice of the background spectrum is not trivial. Usually, classical approaches assume a white noise null-hypothesis to evaluate spectral peaks significance. This would imply that all frequencies are equally important in a time series [55–58], leading to misleading results in natural systems, such as the hydrological ones, whose intrinsic inertia leads to greater power at low frequencies, even in the absence of any signal [59]. It is well known, in fact, that the spectra of measured climatic variables exhibit a general behavior with decreasing power for increasing frequency, rather than being nearly constant [57].

This work aims to integrate classical time-domain modeling approaches with advanced frequency-domain spectral techniques to describe a nonlinear stochastic karst system. By merging complementary information coming from both domains results, the proposed data-driven approach, aims to better characterize the karst system low-term trends dynamics by extracting the significant low frequency variability (LFV) components of meteoclimatic and hydrometeorological variables, to improve the discharge annual forecast in terms of maximum expected variability, while reducing noise propagation and strengthening overall statistical confidence.

To this purpose, we combined multi-regression linear models with the non-parametric Singular-Spectrum Analysis filtering methodology (SSA, hereafter) and its derived gap-filling, statistically significance, noise reduction and forecasting tools [59–74]. SSA provides data-adaptive filters, to decompose a signal into its statistically significant and independent components (i.e., trends, periodic and quasi-periodic oscillations), allowing for both frequency- and amplitude-modulation. This overcomes the limitations of classical spectral methods, which rely on sinusoidal basis functions with fixed frequency and amplitude. SSA offers other advantages with respect to classical Fourier analysis. First, Monte-Carlo SSA (MC-SSA) provides a robust method for assessing the significance of the extracted components from both white and coloured background noises [65,73]. Indeed, natural systems – including hydrological systems and, more broadly, climatic systems – behave as stochastic processes in which long-term variability is amplified, leading to more energy (power) at lower frequencies [59]. Second, SSA provides a gap-filling tool suitable to reconstruct only the significant components of a signal, thereby improving the signal-to-noise ratio without losing information [70,71]. This represents an improvement over classical interpolation techniques, as well as machine-learning-based approaches [41,75], since SSA gap filling respects the whole process average temporal variability and not only the strictly local one.

If the application of SSA in hydrology is known [74,76], the use of SSA-AR (Singular Spectrum Analysis-Autoregressive [63]) forecasting method is still missing in hydroclimatic literature. SSA-AR method is applied in this study to forecast the LFV of the spring discharge with the aim of assessing its maximum ranges of variability by providing a noise reduction strategy.

Particular attention is posed in this research work to the technological transfer of results to stakeholders and local managers. The objective of this study is not to develop a universally optimal physical model of karst systems, but rather to propose a statistically robust, intuitive and reproducible analytical protocol for supporting groundwater management under climate change scenarios. The approach is adaptive, as it regularly updates input parameters whenever new meteorological and discharge data become available, ensuring operational usability for stakeholders and local water managers.

Taking into account what described above, the novelty of the framework lies in the integration of multivariate linear regression modelling with SSA tools within a unified time- and frequency-domain analytical scheme, designed to capture the long-term trend variability in a karst spring discharge. In this framework, the methodology should therefore be regarded as a structured analysis workflow tailored to data-limited karst environments and to operational water-management context, rather than a standalone mathematical model.

The methodological workflow can be applied to other karst aquifers, provided that it is recalibrated using the site-specific hydro-climatic and discharge data. The case study regards a karst aquifer system of the Apuan Alps (northwestern Tuscany, Italy) and the analysis is focused on water resources destined for drinkable water distribution and aims to extract possible empirical relationships between meteorological parameters and groundwater quantity indices. The research is transdisciplinary since it was developed from its preliminary phases in collaboration with stakeholders and local managers, to focus the work from the outset on the technological transfer of the results and methodologies adopted.

## Materials and methods

### Study site: the Cartaro spring in the Apuan Alps aquifer system

**Fig 1:**
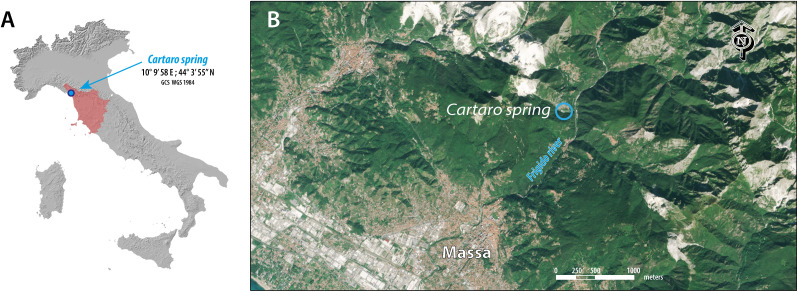
(**A**) Cartaro spring catchment location in the Massa-Carrara province (NW Tuscany, Central Italy); (**B**) Detail of the Cartaro spring catchment and Frigido river inside Apuan Alps. Data sources: Copernicus Sentinel-2 imagery and EuroGeographics Open Data (Open Maps for Europe), used under open data licenses compatible with CC BY 4.0.

The study site is located in northwestern Tuscany (Central Italy, Fig 1A blue circle), a coastal mountain range strongly influenced by Mediterranean climatic conditions and by the proximity of the Ligurian Sea. The relief acts as an efficient orographic barrier for humid air masses coming from the Tyrrhenian–Ligurian basin, producing some of the highest precipitation values recorded in central Italy. According to regional climatological analyses provided by the LaMMA (Environmental Monitoring and Modeling Laboratory) Consortium, mean annual precipitation generally exceeds 1600–2000 mm and can locally reach even higher values at higher elevations. Rainfall is mainly concentrated in autumn and late winter–spring, with monthly rainfall frequently exceeding 150 mm, while summer months are typically characterized by drier conditions [77]. Mean annual air temperature ranges approximately between 10 and 14 °C depending on elevation. These climatic conditions, together with the extensive carbonate formations that characterize the Apuan Alps, favor rapid infiltration and recharge processes typical of karst aquifers [17].

The study site belongs to the Apuan Alps aquifer system, which develops in the metamorphic carbonate sequence of the Apuan Unit. For a detailed description of the Apuan Alps aquifer system in terms of geological, hydrogeological and geochemical settings, refer to the many previous studies devoted to the subject [28,78–82] and references therein. The Apuan Alps aquifer is characterized by poor fracture development at depth, promoting strong inhomogeneity of the groundwater circulation, thus affected by the karst environment. As a result, groundwater mostly flows within well-developed conduit networks, the arrangement of which is driven by friable regime fractures and mother faults. Moreover, superficial fracturing – linked to discharge and physicochemical processes – is responsible for high rates of precipitation infiltration. All these features accentuate the “karstic” character of the Apuan Alps aquifer system which is clearly impacting the hydro-physical behavior of springs.

The Apuan Alps aquifer presents more than 80 springs, whose discharges range on average from 10 to 1600 L/s, most of them with a high variability index [28]. This research work is based on the data collected at the Cartaro spring, located in the basin of the Frigido river (Fig 1B) at an elevation of about 225 m a.s.l. in a channel that descends from the right just upstream from Canevara village. Cartaro spring – characterized by an average discharge of about 400 L/s and a variability index of about 0.831 – was chosen as study site since it is the main source of drinking water supply for the province of Massa-Carrara (Tuscany), despite the unsolved problems associated with the presence of intense mining activity throughout the supply area, which poses continuous risks of pollution [79], making it particularly exposed to climatic change.

### Experimental dataset

#### Meteoclimatic data.

The meteoclimatic dataset was elaborated referring to the main recharge area of the spring. Both precipitation (*P*) and temperature (*T*) data refer to the *2002-Jan/2020-Dec* temporal period and were structured in a GeoTIFF raster format with a degree resolution of about *7.5* arcseconds. The meteoclimatic dataset was created by mining the daily average value of the main recharge area (about *8.4* km^2^) of the Cartaro spring from available database of meteorological parameters spatialized over the Tuscany region at LaMMA Consortium. The spatialized meteorological parameters used in this study are the cumulative daily precipitation and the average daily air temperature obtained as the average of the daily minimum and maximum temperatures which are the available spatialized variables. The daily spatialized data were calculated using all the meteorological daily observations available for the Tuscany Region archived in the LaMMA database for the period *2002–2020*. The weather stations used belong to the networks of Servizio Idrologico della Regione Toscana, Regione Liguria ed Emilia-Romagna, Aeronautica Militare, LaMMA Consortium, UCEA, and other minor networks for a total of *1169* pluviometric stations and *680* thermometric stations.

The spatialization was performed using an improved version of the Daymet algorithm [83]. This algorithm generates a spatial interpolation of the meteorological variables using a DTM of the area of interest and the observations from a series of weather stations. The original algorithm has been calibrated for the Tuscany region, using a DTM at *250* m around Tuscany (in the area between *44.5719–42.1323* ° N latitude and *9.68646–12.474* ° W longitude) for the period *1995–2011* (*17* years). The results of cross-validation for the calibration period show a mean error of *0.0074* mm, *−0.0474* °C and *0.0239* °C and mean absolute error of *1.3601* mm, *1.3521* °C and *1.1063* °C for the daily precipitation, minimum and maximum temperature, respectively.

Meteoclimatic data are reported in S2 File Temperature *T* is expressed in [° C], precipitation *P* in [mm].

#### Discharge data.

With the support of AIT (Tuscan Water Autority), spring discharge (*Q*) data were collected from water managers (of the study area) and the related technical facilities, in particular GAIA SpA Integrated Water Service. The Cartaro spring discharge time series covers the period from *2002-Jan to 2020-Dec* at a daily temporal resolution. For each day the average discharge of the Cartaro spring was calculated from daily cumulative volumes, i.e., the volumes delivered by the Cartaro source in over the reference time (day). Discharge data *Q* are reported in S2 File and are expressed in [L/s].

### Data analysis

#### Descriptive statistics and cross-correlation analysis.

Basic statistical analysis was performed to characterize the main features of the dataset over the study period, including average values, standard deviations (*std*), minimum values (*min*), first quartiles (*Q*_*1*_), median, third quartiles (*Q*_*3*_), maximum values (*max*). Cross-correlation analysis was used to investigate the temporal relationships between meteorological variables (*T* and *P*; independent variables) and spring discharge (*Q*; dependent variable) by computing the correlation coefficient at *lag = 0* and across different time lags to account for the dataset temporal reciprocal variability. Cross-correlation results for *lag = 0* are shown by classical cross-correlation matrix chart which can be interpreted as it follows: the distribution of each variable is shown on the chart diagonal; the bivariate scatter plots are reported on the bottom of the diagonal; finally, the correlation coefficients (by Pearson) are displayed on the top of the diagonal together with the corresponding significance level, indicated as stars in accordance with the following convention *** = P≤ 0.001, ** = *P≤ 0.01*, * = *P≤ 0.05.* The R “stats” package was used to compute statistical functions (https://stat.ethz.ch/R-manual/R-devel/library/stats/html/stats-package.html).

#### Advanced spectral analysis and gap-filling by singular spectrum analysis (SSA).

The dataset was filtered by singular spectrum analysis (SSA) [60–74] to extract its frequency and amplitude-modulated oscillatory components (both periodic and quasi-periodic) and trend from a suitable background noise. SSA is an advanced spectral analysis technique designed to get information about non-linear systems from short and noisy time series without appealing to the process governing equations, by providing data-adaptive filters which allow the decomposition of time series into statistically independent components or reconstructed components (*RCs*). Monte Carlo SSA (MC-SSA) was used to perform signal-to-noise (S/N) separation against a red-noise null hypothesis [65,73]. MC-SSA allows to overcome the limitation of the traditional denoising approach (i.e., the identification of a noise floor in a sequence of eigenvalues given in descending order) which becomes problematic when either the S/N ratio is not sufficiently large or the noise affecting the data is not simply white but “coloured” [59], such as in our case.

The gap filling version of SSA was applied to process the flow time series, which had uneven sampling due to missing observations [70,71]. In the SSA frequency-domain gaps are filled-in by using the temporal correlations, thus accounting for the true signal embedded into the analyzed time series, only. With respect to traditional methods (e.g., linear fit and moving average), this approach respects the whole process temporal variability and not only the strictly local one (i.e., near the gap). Gap filling was performed by setting SSA window size to *M = 2420* (*<*⅓ of the total series length) and the number of significant components to *C = 30*. These parameters were chosen to cover the largest temporal correlations but, at the same time, to retain as much signal as possible without introducing random noise into the signal gap-filled reconstruction. Refer to Appendix *Singular spectrum analysis (SSA)* for more details about both denoising and gap-filling procedures. To perform SSA analysis, the SSA-MTM Toolkit (http://www.spectraworks.com/web/welcome.html [58]), as well the UCLA freeware toolkit at http://research.atmos.ucla.edu/tcd/ssa/guide/guide4.html#disclaimer [59] were used. To assess the potential influence of the gap-filling procedure on the modelling results, a sensitivity test was performed by reconstructing missing values also using simple linear interpolation and comparing the two gap-filled time series. The nonparametric Kolmogorov-Smirnov (KS) test was performed to assess whether the two series were drawn from the same underlying probability distribution (null hypothesis, H0). Since the *P-value* of the KS test was greater than 0.1, the null hypothesis was not rejected.

#### Multi-regression linear models.

Multi regression models were built as explanatory tools to infer causal relationships among the dependent variable and multiple regressors (or independent variables). In our case, the dependent variable is karst spring discharge *Q*, while the regressors are the meteoclimatic variables, i.e., temperature *T* and precipitation *P*, or delayed/averaged versions of the same. Before building the models, a linear partial correlation analysis was performed to account for spurious correlations due to eventual regressors collinearity. Partial correlation was achieved as a linear correlation between pairs of regressors (ρ_part_), fixing the remaining independent variables [84–86]. Only those regressors showing a statistically significant correlation with the dependent variable, and – at the same time – a low correlation with all the other explanatory variables, were included in the model. For this purpose, MatlabR2020a *partialcorr* function was used, as well as its Python clone script at https://gist.github.com/fabianp/9396204419c7b638d38f.

The model's in-sample performance was evaluated using several statistical indicators. Model accuracy was assessed through the coefficient of determination (*R²*), while the statistical significance of the overall model fit and of individual predictors was determined using *P‑values*. Several models were tested and the most suitable one was selected according to the Akaike Information Criterion (AIC) [87]. Results from the out-of-sample multivariate regression will support the selection and characterization of the forecasting parameters adopted in the spectral methods, in particular the SSA filtering window and the number of significant components to forecast (§ *Time series prediction: SSA‑AR method*).

All the scripts used for performing linear models were written in Python code using libraries *numpy*, *pandas*, *matplotlib*, and *statsmodels*.

#### Time series prediction: SSA-AR method.

Single-channel time series prediction was performed on the discharge and was based on the combination of classical low-order autoregressive (AR) models [88–90] and singular spectrum analysis (SSA) as an innovative noise reduction forecasting strategy [63,91,92]. The order of the AR model was set equal to the SSA filtering window (*M*) and it was fine-tuned using the time-domain analysis results, as well as the number of LFV components to forecast (*C*). Refer to section *SSA-AR prediction* in the *Appendix* for more details about the methodology. In this paper, AR-SSA methodology was applied to a shorter version of *Q (N =* 3652*)* to ensure the comparability with the multi-regression model’s approach. Time lapse *T*_1_= [2010,2019] was used to predict one more year (2020). The order of the autoregressive process, as well as the SSA filtering window, were chosen as *M*_AR_ ~ *M*_SSA_ = 500. The prediction of all the significant *RCs* was yielded over a maximum lead time of one year, *L*_max_ = 365, in agreement with the multi-regression linear model’s approach. Learning and test sections lengths were set to *N*_test_ = 1787 and, *N*_learn_ = 1500, respectively, while the sliding window length was set to *K* = 1422. The predictions of all the RCs were summed to yield the total prediction over *Q*_pred_, and the corresponding uncertainty was computed as the square root of the sum of the squared RMS errors of the individual RC forecast.

Forecasting and cross validation were performed by using the toolkit available at http://www.spectraworks.com/web/welcome.html (refer to [69] and references therein).

## Results

To assess the relationship between meteoclimatic variables and Cartaro’s spring discharge, the experimental dataset was first analyzed by standard cross-correlation analysis (section *Descriptive statistics of the dataset*) and multi regression linear models (section *Multivariate regression modeling*). The results obtained using these two analytical methods were combined with those from the SSA, to finally set the parameters of the SSA-AR forecasting procedure, based on the extraction and prediction of the dataset low-frequency variability (LFV) to limit error propagation during forecasting and identify the maximum range of discharge variability (sections *Spectral analysis and extraction of the significant components* and *SSA-AR forecasting*).

### Descriptive statistics of the dataset

The descriptive statistics of temperature (*T*), precipitation (*P*) and discharge (*Q*) time series is reported in Table 1.

**Table 1:** Descriptive statistics of the analyzed variables: discharge (*Q*), precipitation (*P*) and temperature (*T*). The corresponding units of measurement are given in the header. The following basic statistics are reported for each variable: average, standard deviation (*std*), minimum value (*min*), first quartile (*Q*_*1*_), median, third quartile (*Q*_*3*_), maximum value (*max*), and total number of samples (*N*). For *Q* the number of gaps is also indicated. Study period: 2002-Jan/2020-Dec.

	*Q [L/s]*	*P [mm]*	*T [°C]*
*average*	276.07	4.80	11.98
*std*	82.75	12.73	6.60
*min*	89.72	0	-6.54
*Q_1_*	208.9	0	6.68
*median (Q_2_)*	284.04	0.05	11.62
*Q_3_*	342.73	2.64	17.18
*max*	514.58	209.41	28.96
*N*	6666 (1077 gaps)	6666	6666

**Fig 2:**
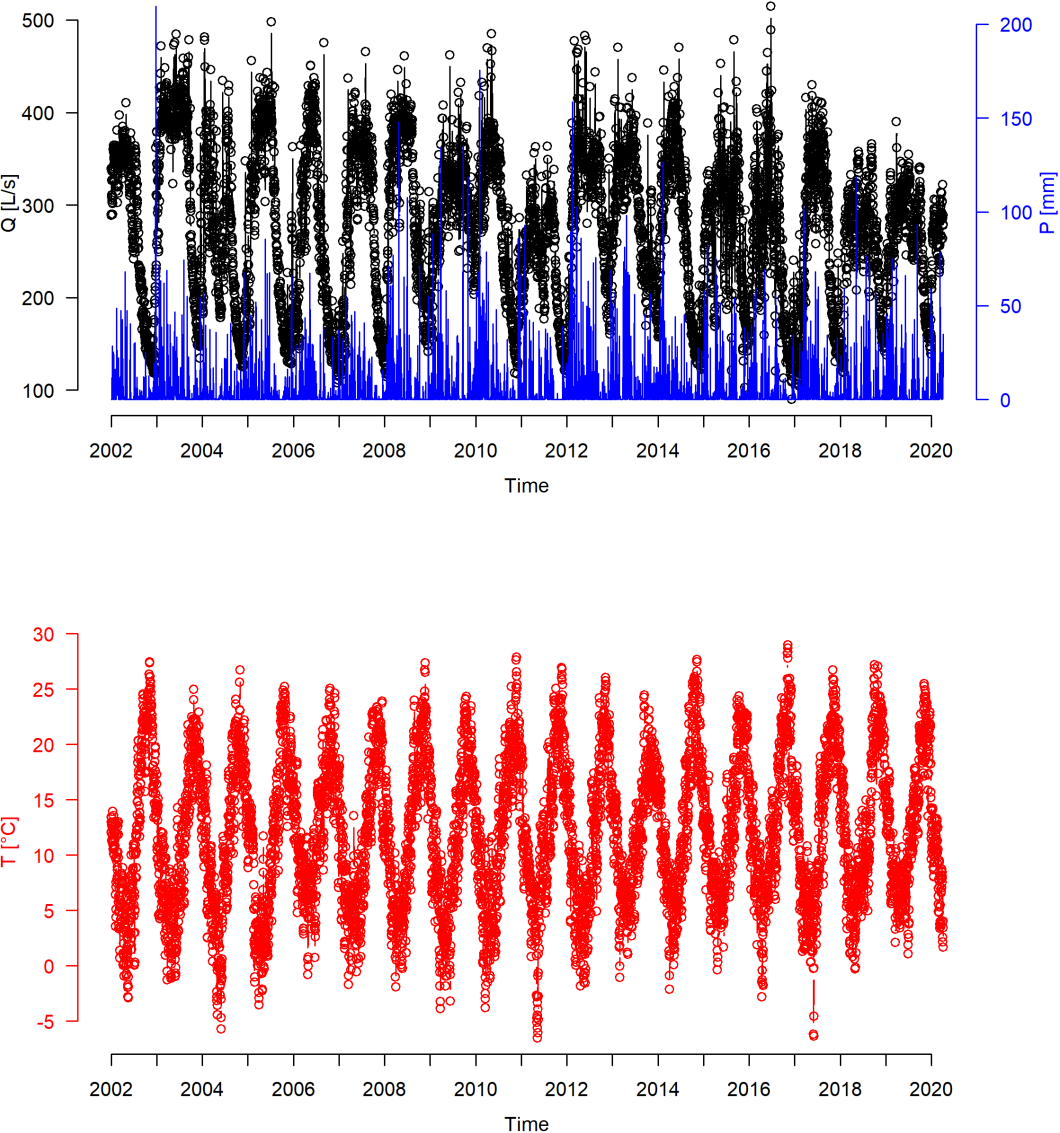
(A) Cartaro spring discharge (*Q*, left axis) and precipitation (*P*, right axis) time series. (**B**) Temperature time series (*T*). Study period: 2002-Jan/2020-Dec.

SSA-gap filling was required for the Cartaro discharge time series, since *1077* gaps were present out of a total of *N = 6666* samples (*~16%*) due to probe malfunction caused by heavy rainfall. Instead, no gap filling was necessary for *P* and *T*, which were evenly spaced series by construction. The gap-filled discharge (*Q*) is reported in Fig 2 (panel A, left axis) together with precipitation (*P*, panel A, right axis) and temperature time series (*T*, panel B).

**Fig 3:**
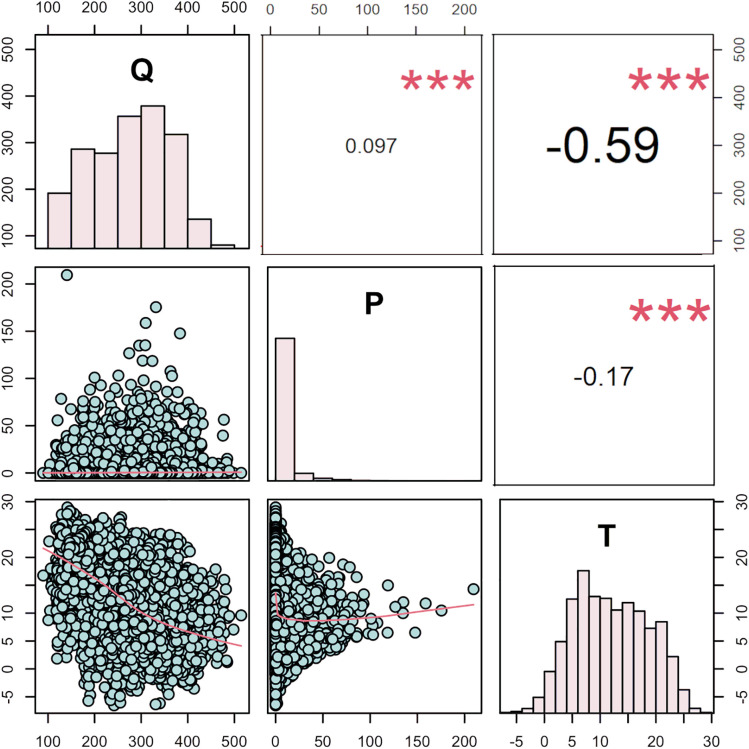
Correlation matrix (lag 0) between *Q*, *P*, *T.* The paired correlation coefficients (by Pearson) are displayed together with the corresponding significance level, indicated as red stars in accordance with the following convention *** = P ≤ 0.001, ** = P ≤ 0.01, *P ≤ 0.05. Upper panels text size is proportional to the correlations. Study period: 2002-Jan/2020-Dec.

Fig 3 shows the correlation matrix charts among couples of variables at lag = 0. A strong and negative correlation emerged between *Q* and *T*, while low correlation was observed between *Q* and *P*. Finally low (linear) correlation was detected between variables *P* and *T*. The same relationships were confirmed by partial correlation analysis: A negative correlation was found between *Q* and *T* (*ρ*_*partQ,T*_ = −0.46***), while no correlation was found between *Q* and *P* (*ρ*_*partQ,P*_ *= −0.02*). The dependency (at least linear) of *P* and *T* was confirmed (*ρ*_*partP,T*_ = 0.20***).

Present results suggested the need to introduce time lags in the cross-correlation analysis to catch the real dependency between *Q* and *P.*

### Multivariate regression modeling

Transformed versions of the input variables were used to maximize the correlation coefficients of precipitation with discharge, at the expense of temperature. Assuming a delayed reaction of aquifer recharge to the meteo-climatic inputs, cross-correlation analysis was repeated over both lagged and averaged (based on running mean) versions of *P* and *T* (refer to the *Supplementary Material*, § *S.1 Cross-correlation analysis* in S1 File).

From cross-correlation analysis, three main classes of models were identified, depending on the transformation applied to raw data (Tables S1.1, S1.2, S.1.3 in S1 File). All models were fitted on the interval Jan 2016-Dec 2019 (in-sample), while the out-of-samples forecasting was performed on a one-year interval (Jan2020-Dec2020).

For each class, the best fit was chosen by the AIC test performed on the in-sample interval. Table S2.1 in S1 File shows, for each class of models, the best fit equation. The whole ordinary least squares (OLS) regression results are reported in Tables S2.2, S2.3, S2.4 in S1 File, for the three classes respectively. For all the models reported in Table S2.1 in S1 File, both the *P*-value of the chosen models, and the *P*-value of each regressor turned out to be significant at the 99% c.l. (*P < 0.001*).

**Fig 4:**
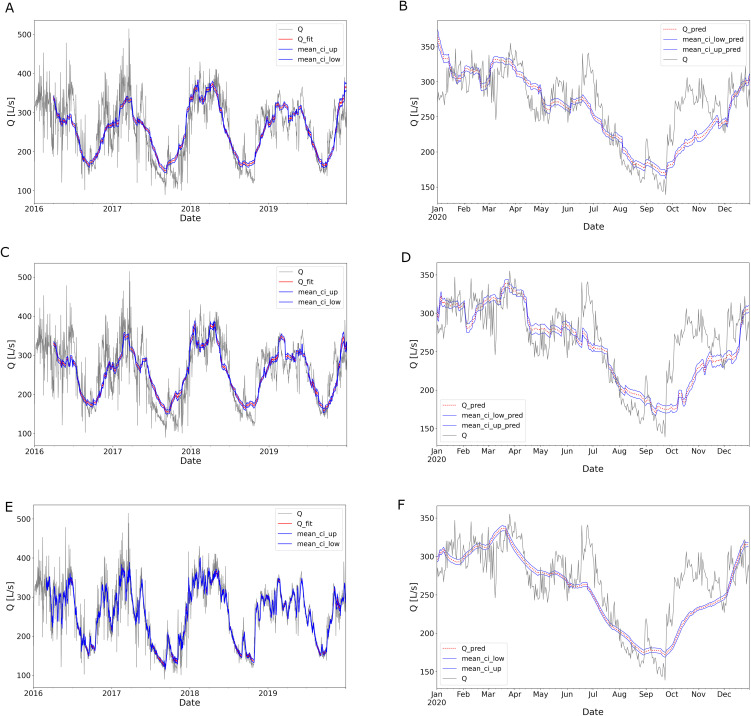
Upper panels. Class 1 model. In sample prediction (**A**) and out-of-samples prediction (**B**). Best fit: Q ~ P_m60 + T_m45 + T_m90. Medium panels: Class 2 model. In sample prediction (**C**) and out-of-samples prediction (**D**). Best fit: Q ~ P_m30_L15 + T_m30_L30 + T_m30_L60. Lower panels: Class 3 model. In sample prediction (**E**) and out-of-samples prediction (F). Best fit: Q ~ Q_m5 + P_m20 + T_m60. In all the cases *m* represent the days included in the rolling means and *L* the time shift of the time series. Fitted values are shown in red, and confidence intervals in blue. Raw data are shown in gray. Model fit in-sample section: Jan2016-Dec2019; out-of-sample forecasting period Jan2020-Dec2020.

Fig 4 shows, for all the three classes of models, the best fit in-samples predicted values (red line, panels A, C, E for class 1, 2, and 3, respectively) together with the 95% confidence intervals of the mean (blue lines), and the best fit out-of-samples mean prediction (red line, panels B, D, F for class 1, 2 and 3, respectively) together with the 95% confidence intervals over the mean predicted value (blue lines).

In the following, the three classes of models are explained into detail.

#### Short-term memory (Class 1).

The first class of models uses smoothed versions of *P* and *T*, obtained as rolling mean computed over *m*-past values (daily units), thus emphasizing the short-term memory of the system.

Cross-correlation analysis (Table S1.1 in S1 File) limited to the in-sample interval highlighted that a simple shift of the precipitation series was not sufficient to improve its correlation with the discharge. Rather *c*_*Q-P*_ increased by considering the rolling mean computed over the last *m* values of the precipitation. Increasing *m* from *20* to *90* (< 3 months) the correlation coefficient increased, while for *m > 120* (> 4 months) it reached an upper limit (*ρ*_*Q-P,m>90*_ *= 0.71*). This suggests that past precipitation regimes, from twenty days to three months, can influence spring discharge at our site. Moreover, the heavy-tailed distribution of precipitation time series – evident from both Fig 3 and Table 1 – implies to consider filtered or smoothed versions of *P*. The correlation between *Q* and *T* improves for growing *m*, reaching an upper limit when *m > 90* (3 months). Cross-correlation analysis suggests that after three months, the influence of *P* and *T* on *Q* appears to be negligible.

Taking into account cross-correlation results, the model best fit (Tables S2.1 and S2.2 in S1 File, Fig 4A-B) was built. Its coefficients suggest a strong positive relationship between *Q* and past averaged *P* values and a positive or negative relationship between *Q* and past averaged *T* values, depending on the time shift. This is due to the strong oscillatory behavior of the temperature time series which *Class 1* models cannot fully catch.

#### Long-term memory (Class 2).

The second class of models uses smoothed and delayed versions of *P* and *T* obtained as rolling mean computed over *m*-values taken at *L* time step backwards (daily units), thus emphasizing the average long-term memory and the cyclic behavior of the system.

Cross-correlation analysis limited to the in-sample interval (Table S1.2 in S1 File) confirmed the previous results: A simple shift in precipitation was not sufficient to explain *Q* variability (*ρ*_*Q-P*_ *< 0.2*). The correlation improves if the mean over *15−30* days – taken at different lags *L* – is computed. The maximum *Q-P* correlation is reached for a shift *L = 15* days and a *30*-days rolling mean (*ρ*_*Q-P,max*_ *= 0.54*), while the maximum *Q-T* correlation is reached for *L = 30* days and a *30*-days rolling mean (*ρ*_*Q-T,max*_ *= −0.74*).

Model best fit and related coefficients (Table S2.1 and Table S2.3 in S1 File, Fig 4C-D) allowed to improve *Class 1* models results interpretation: the relationship between *T* and *Q* is characterized by a negative coefficient, in line with the fact that *Class 2* model uses the temperature monthly averages computed for two different *L* shifts, thus better accounting for the dominant cycles embedded into the temperature time series. Also in this case, the precipitation occurring on average in the last 15–20 days has the greatest influence on the spring's discharge.

In both *Class 1* and *Class 2* models a shift was present between real and fitted values, both in the in-sample fit section (Fig 4 A and C) and in the out-of-samples prediction (Fig 4 B and D), despite generally good agreement. This means that *Class 1* and *Class 2* predictors are not sufficient to fully represent the spring behavior, and that an additional parameter connected to the aquifer structure itself needs to be introduced.

#### Autoregressive term (Class 3).

The last class of models aims to overcome this problem by introducing the discharge itself among the previous regressors to account for an autoregressive term able to represent the state of the karst system at earlier times and, thus, its intrinsic characteristics. This term recalls the presence of a memory effect, well documented in karst aquifers due to their complex storage and flow dynamics [93,94].

Here the memory term is introduced into the model in an operational way, without explicitly resorting to the governing equations of the physical process.

Cross-correlation analysis over the in-sample section (Table S1.3 in S1 File) shows that Cartaro karst system has a short memory of about 5 days (*ρ*_*Q-Qlag,max*_ *= 0.89*). Considering larger temporal lags, the correlation decreases. The best fit (Table S2.1 in S1 File, Fig 4E), built from cross-correlation analysis, shows a clear improvement over the in-sample section reaching *R*^*2*^ *~ 0.8* (Table S2.1 in S1 File, Fig 4E). The annual predicted discharge over the out-of-samples section can also reproduce the short-term variation and not only the long-term trend (Fig 4F). This suggests considering the short-time memory of the discharge in next analyses by spectral methods. The progressive improvement observed from *Class 1* to *Class 3* models, in fact, highlights the importance of accounting for the intrinsic memory of karst systems, related to low-frequency variability.

### Sensitivity analysis of multivariate regression models

Changing the temporal length of the fit period (e.g., reducing it to Jan 2018-Dec 2019), the cross-correlation results slightly change (Table S1.4 in S1 File), as well as the temporal interval on which it is necessary to consider rolling means and variable shifts to maximize the explained variance. Despite variations in both the regression coefficients and the goodness of fit, the overall structure of the model remains unchanged (Table S2.1, model class: 3b; Table S2.5 in S1 File). Therefore, the results of multivariate regression models must be understood as both site- and time-dependent since the Cartaro karst system shows great variability over time, which is amplified by feedback effects related to the huge exogenous input represented by climate change forcing. Nevertheless, these methods are suitable to detect the long-term variations of the Cartaro’s system.

### Spectral analysis and extraction of the significant components

Previous time-domain results were integrated with advanced spectral analysis. In particular, they were used to fine-tune the SSA filtering window (*M*) and the number of significant components (*C*) to retain for further analysis, including LFV forecasting. We refer to the sections *Advanced spectral analysis and gap-filling by singular spectrum analysis (SSA)* and *Time series prediction: SSA-AR method* in *Materials and Methods* an*d Singular spectrum analysis (SSA) and SSA-AR forecasting* in the *Appendix* for more details about the analysis settings.

SSA method was applied to extract the dataset’ s low frequency variability (LFV) with respect to a suitable noise background to:

i better estimate the significant contribution of the meteoclimatic variables to the discharge temporal evolution;
ii perform the long-term forecast (half-yearly or annual) of the discharge using the combined SSA-AR methodology, considering only its low frequency significant components and thus reducing noise propagation.

**Fig 5:**
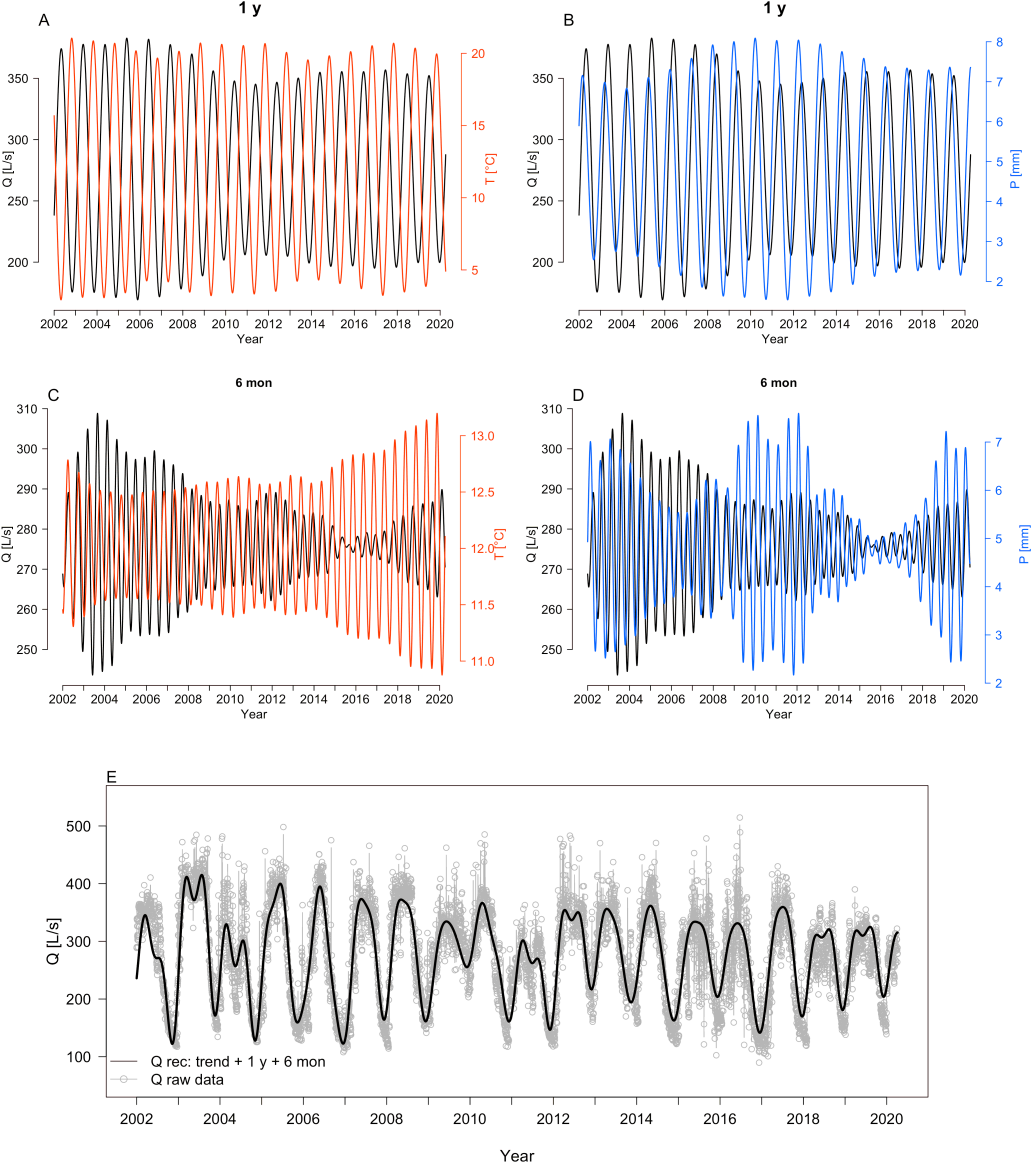
(A-B) Annual cycle revealed in the karst spring discharge *Q* (black line), temperature *T* and precipitation *P* (panel A-red line and panel B-blue line, respectively). The annual periodicities turned out to be significant at the 99% c.l. in all variables. (**C-D**) 6-month oscillation detected in *Q* (black lines), *T* (panel A, red line), and *P* (panel B, blue line). The semestrial cycle was significant at the 95% c.l. in all the time series. It became significant at the 99% c.l. after the removal of the annual cycle. (**E**) SSA reconstruction of the discharge low-frequency variability (black bold line) superposed to *Q* raw data (gray). Study period: 2002-Jan/2020-Dec.

SSA results are shown in Fig 5 and suggest that both discharge and meteoclimatic variables are dominated by annual and semi-annual significant cycles. While such periodicities are commonly observed in hydrological signals, the SSA decomposition also highlights the modulation of their amplitude through time, reflecting the influence of low-frequency variability in the recharge dynamics of the karst system. Higher frequency signals (i.e., shorter periods at weekly or daily scale) were not considered during this study, since beyond the focus of the work.

Fig 5 shows the annual cycle extracted from *Q* (black line in both panels A and B) *T* (red line, panel A) and *P* (blue line, panel B). The 6-month cycle detected in *Q* (black line in both panels C and D) is shown with the corresponding semi-annual components detected in *T* (red line, panel C) and *P* (blue line, panel D).

The annual periodicity – significant at the 99% c.l. – is dominating *Q* and *T* low frequency variability, and it accounts for the 55% and 80% of the series total variance, respectively. After detrending by annual oscillation, the semestral cycle turned out to be significant at the 99% c.l., accounting for the 15% and 5% of *Q* and *T* signals total variance, respectively.

As expected, the situation is different for *P*, whose cyclic components are accounting for less than 5% of series total variance, possibly owing to the heavy-tailed behavior of the precipitation probability density function (Fig 3).

The total SSA reconstruction of discharge’s LFV is shown in Fig 5E (bold black line) superposed to raw data (gray). The process low-frequency dynamics are quite well represented by the SSA reconstruction, explaining about 60–70% of the series total variance. Analogously to the time-domain approach, SSA highlights the presence of a residual non-significant signal, especially in the high frequency range (i.e., shortest periods).

### SSA-AR forecasting

Starting from LFV reconstruction by SSA and time-domain results, discharge forecasting was finally computed by predicting separately each of the narrowband signals contained in *Q*, namely the annual and semiannual cycles shown in Fig 5E, and residual low-frequency component (f_LF_ < 0.01 day^-1^). Time lapse *T*_1_= [2010, 2019] was used to predict one more year (Jan-Dec 2020). To conform to the approach used for regression models, SSA-AR forecasting was also performed using a shorter time lapse *T*_2_= [2015, 2019] for the learning and test sections. The maximum lead was always set to one year.

**Fig 6:**
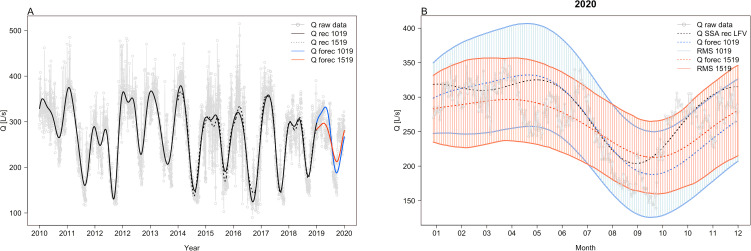
(A) Discharge significant reconstructions of LFV over *T*_*1*_ (1019 solid black line) and *T*_*2*_ (1519 dotted black line). The corresponding annual predictions are shown from 2020 for *T*_*1*_ (1019 blue line) and *T*_*2*_ (1519 red line), respectively. (**B**) Enlargement over the forecasted year (Jan-Dec 2020). Both forecasted discharge and the corresponding forecasting uncertainties are shown for two different learning-test sections *T*_*1*_ (red) and *T*_*2*_ (blue). SSA reconstruction of LFV is also reported (black).

Fig 6A shows SSA-AR prediction results. The SSA low frequency reconstructions are shown for both *T*_1_ (*Q*_*rec,1019*_, solid black line) and *T*_2_ (*Q*_*rec,1519*_, dotted black line). Both SSA reconstructions fully reproduce discharge LFV, and no temporal delay is present between raw and reconstructed time series, thanks to SSA filter data-adaptability. The corresponding discharge annual predictions for 2020 are shown as red (*Q*_*forec,1519*_) and blue (*Q*_*forec,1019*_) lines, while the forecast uncertainties are shown with the same color scheme in Fig 6B, where also the true LFV SSA reconstruction is reported (black dotted line).

With respect to the results found by linear regression models, forecasting uncertainties are larger, as expected, since only the discharge LVF is projected. In general, both SSA-AR predictions show a good agreement with the discharge’ LFV reconstruction in the first semester, especially for larger training sections (*Q*_*forec,1019*_). Starting from month 9, AR-SSA forecasting underestimate real values. The maximum range of discharge variability, is well represented for shorter training sections, owing to the non-linear dynamics and variability of the karst system. Overall, SSA-AR forecasting yields more realistic results in terms of the range of variability compared to forecasting using regression models.

## Discussion

This work focuses on the study of an Italian karst aquifer (Cartaro spring in Apuan Alps, Tuscany) in the context of climate change. The aim of this research is to introduce a decision-support analytical protocol to support decision makers in groundwater management, by providing suitable long-term forecast (half-yearly or annual) of the average behavior of the Cartaro’s spring discharge in a transdisciplinary perspective. To this purpose, an innovative data-driven approach, based on the simultaneous application of time-domain (multivariate linear regression) and frequency-domain methods (Singular Spectrum Analysis) – was proposed. The final goal was to characterize and forecast the low frequency variability (LFV) of the Cartaro’s spring discharge and its maximum range of variability. Unlike traditional methods, particular attention has been paid to reduce noise propagation.

**Fig 7:**
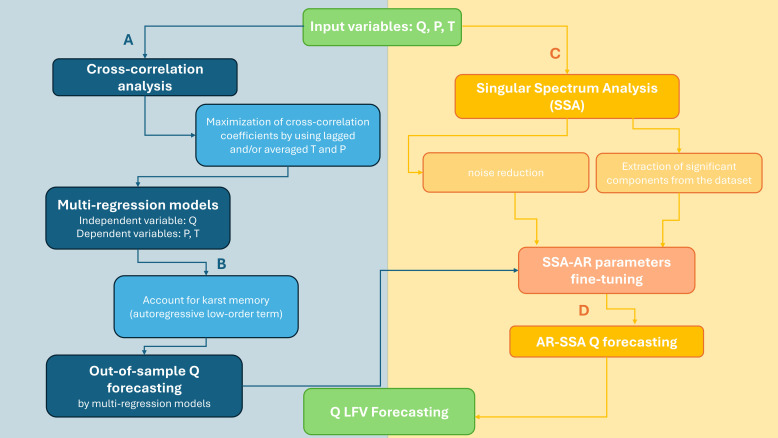
Workflow for prediction of Cartaro’s spring discharge LFV. On the left are reported the time-domain approaches (blue), on the right the frequency-domain ones (orange). Time-domain and frequency-domain methods are combined to set the forecasting parameters (red).

The analysis protocol, is shown in Fig 7. Time-domain methods are reported on the left (blue), while the frequency-domain techniques are reported on the right (orange). The protocol steps are summarized in the following paragraphs.

### Hydrological interpretation of the regression models

First time-domain methods – cross-correlation analysis and multi-regression models (Fig 7A, left section in blue) – were used to infer causal relationships between Cartaro discharge and the meteorological variables affecting the study area. This preliminary analysis revealed the following:

i A strong anticorrelation between the discharge and the temperature, which should be interpreted in strictly local seasonal terms, i.e., in Apuan Alps it rains more in the cold season, in agreement with local climatological data which indicates that the Apuan Alps experience their highest precipitation during the cold season (autumn–winter), with monthly rainfall frequently exceeding 150 mm [77]. Moreover, both close and far (lagged) temperature values influenced the discharge, indicating an intertemporal compensation in line with the presence of oscillatory components within temperature and discharge time series which cannot be neglected.
ii An apparent lack of correlation between *Q* and *P*, in disagreement with reality, owing mainly to: *(i)* The high degree of non-stationarity in precipitation, which clearly follows an heavy-tailed distribution (Fig 3) differently from *Q*. This is due to the spike-like behaviour of Apuan Alps rainfall, which is dominated by very intense rainstorms events concentrated within a few hours [95,96]; *(ii)* The need for cumulative precipitation events, rather than isolated precipitation peaks, for the karst spring to recharge. In particular, the analysis suggested that past precipitation can influence spring discharge dynamics starting from twenty days on. This result agrees with karst literature which bases on cross-correlation analysis the study of the relationships between precipitation and discharge [29,51–54]. Many studies have shown that karst springs do not respond to isolated rainfall spikes, but rather to cumulative rainfall over extended periods, which can last up to several months, depending on the spring [96,97].
iii Classical spectral analysis has suggested the presence of a memory effect that modulates short-, medium-, and long-term precipitation, based on how internal karst reservoirs fill and empty depending on the level of karstification of the aquifer itself [51].

According to previous results, the discharge analysis was improved, to account for memory effects. The discharge itself was introduced among regressors to represent the state of the karst system at earlier times (Fig 7B). This term must be understood as an additional parameter representing the inherent non-linear dynamic of karst, which in turn is reflected in the discharge as an “infiltration delay” [51] in general estimated to be about 5–20 days. The infiltration delay at Cartaro’s spring is short (less than one week). Thus, the Cartaro karst system is highly dependent on immediately preceding dynamic states, and this dependence is mainly related to precipitation patterns.

Previous results make us confident to assess that the lack of other important parameters in the regressors – such as evapotranspiration – is not the principal cause of discrepancy between raw and modeled discharge. In fact, karst system recharge is often highly focused at sinkholes or stream networks and therefore is generally less dependent on evapotranspiration and soil cover than in other hydrogeologic settings [30]. Moreover, evapotranspiration is largely controlled by climatic variables, particularly temperature and solar radiation, and therefore shows strong seasonal correlation with air temperature. Including both variables in the regression models would introduce significant multicollinearity which can inflate the variance of regression coefficients and reduce the stability and interpretability of the model parameters [98–100]. For this reason, temperature was retained as a proxy climatic variable implicitly accounting for evapotranspiration effects.

Sensitivity analysis revealed that multi-regression models were sensible to changes in the test (fit) section, notwithstanding the general structure of the models remained unchanged. Two main reasons have been hypothesized:

i A change in the system's response to meteorological variables, owing to both sudden changes in the precipitation regime and temperature oscillations, connected with global warming;
ii A change in the internal conduits affecting the underground flow path network of the aquifer owing to its attempt to adapt to different precipitation regimes, local disturbances or structural changes.

### Identification of low frequency variability of karst spring discharge

Time-domain results were used to fine-tune frequency-domain methods parameters, in particular SSA number of significant components (*C*) and the dimensions of the filtering window (*M*). Advanced spectral analysis was performed by SSA to extract the database LFV with respect to a suitable background noise, thus overcoming the limits of classical methods and introducing a new perspective in these studies (Fig 7C). Advanced spectral methods were chosen to support multi-regressions models since they are intimately connected with the nonlinear dynamics of a natural system and suitable to describe not-stationary dataset [57,69].

SSA revealed that both discharge and meteoclimatic variables were dominated by annual and semi-annual amplitude-modulated significant cycles. Although the presence of these cycles is known in the study area, our analysis recall the need to use advanced techniques rather than classical spectral methods. In particular:

i The annual cycles detected in *Q* and *T* are in antiphase, in agreement with previous findings, perhaps due to local/regional features in precipitation regime;
ii Both annual and semi-annual cycles detected in *Q* and *P* are slightly shifted with respect to each other confirming multi-regression linear models results: There is a little temporal delay (about 20 days) between precipitation occurrence and Cartaro karst spring answer;
iii The response of the karst aquifer to similar precipitation inputs is different, confirming a nonlinear input-output relationship of the system.

Differently from regression models, no temporal delay was observed between discharge and its LFV reconstruction, thanks to the data-adaptability of SSA filtering to non-stationary series. This confirms that SSA is effective in isolating meaningful signal components from noisy hydrological time series, an aspect where traditional parametric methods often fail, leading to noise amplification in predictive models [57,72]. The LFV reconstruction was able to capture 70% of the total variance in the series. Similarly to multi-regression models, SSA highlighted the presence of a residual signal (not-significant) especially in the high frequency range. This residual signal (30% of the series total variance) – related to system noise – was filtered before performing further analysis (e.g., forecasting).

### Forecasting capability of the SSA-AR methodology

SSA-AR methodology was applied to forecast the discharge dynamics over a 1-year temporal period, considering only the LFV extracted by SSA (Fig 7D). SSA-AR forecasting complemented and enhanced traditional multi-regression models results by providing a more robust interpretation of the underlying dynamics. Here the main results:

i SSA-AR forecasting was able to reproduce the karst inter/intra annual LFV dynamics losing lower-frequency pattern, which was better represented by previous time-domain models.
ii Prediction showed a good agreement with the real signal LFV reconstruction in the first semester, while for a longer time span AR-SSA prediction underestimated real values.
iii The discharge maximum range of variability was well represented with respect to time-domain modeling, especially if the prediction was trained on smaller sections, owing to the karst system nonlinearity.

These results suggest that there is a trade-off between time-domain and frequency-domain methods. If, as in our case, the goal of the analysis is to predict average discharge behavior and its maximum range of variability, spectral methods are recommended. If short-term variations need to be modeled – primarily those linked to sudden, impulsive precipitation events – time-dependent methods are more suitable, even though they introduce greater noise into the forecasts. The combined application of both domains methods is suggested.

Usually in the literature precipitation issue is solved by cumulating or smoothing this variable, while the heavy-tailed features of its probability density function are not taken really into account. This affects data analysis and introduces further noise into the series which may be the cause of the unexplained signal from previous models [101,102].

### Limitations and transferability of the proposed framework

Overall, the results obtained from our analysis are local. It is not possible and not recommended to look for a generalized model, as perhaps the carbonate system of the Cartaro’ aquifer itself is evolving. Recent studies on karst spring discharge modelling have shown that the performance and transferability of data-driven models are strongly influenced by site-specific hydrogeological conditions and by the availability and representation of hydrometeorological data [35,101–103]. This supports the interpretation of the present framework as locally valid and suggests that future adaptive implementations should rely on periodic recalibration and updating as new data become available.

Although karst aquifers are characterized by strongly nonlinear dynamics, the use of multiple linear regression as a first step in this study was motivated by the need for a transparent and interpretable modelling framework suitable for decision-support purposes. Although more complex nonlinear approaches could potentially improve the representation of the system’s dynamics and could be explored in future studies, we chose to use SSA, which is even better suited to accounting for the system’s nonlinearity.

Finally, the proposed modelling framework was primarily designed to reproduce the average behavior and uncertainties of the spring discharge low-frequency variability, according to water managers and local stakeholders requirements. In particular, the primary objective was to provide a robust representation of long-term fluctuations in water availability, rather than the simulation of short-term extremes. Therefore, the present approach is not intended to capture short-term extreme events, such as peak flows associated with intense precipitation episodes, which were out of the scope of this transdisciplinary collaboration. However, the theory of extreme events can be incorporated into this approach with a view to future developments [104,105].

## Conclusion

This study proposes a decision-support analytical protocol for forecasting the hydrological behavior of an Italian karst spring system (Cartaro spring, Apuan Alps, Italy), combining multivariate regression models and Singular Spectrum Analysis (SSA). The objective of the research was to explore whether a data-driven approach combining time-domain regression analysis with spectral decomposition techniques could effectively describe the complex dynamics of karst spring discharge and support long-term groundwater management. We made this choice so that we could use methods ranging from the simplest linear ones to the most complex spectral ones, thereby gradually capturing the complexity of the nonlinear system without resorting to complicated models that cannot be implemented from a technological standpoint. In fact, the analytical framework was designed primarily to reproduce the average behavior and low-frequency variability of the source’s flow rate, in collaboration with water resource managers and local stakeholders.

The methodology reported here successfully identifies non-linear causal relationships between meteo-climatic variables and the spring discharge, as well as their low-frequency variability, offering reliable annual forecasts of the discharge and its maximum range of variability, while minimizing background noise. This is possible thanks to the SSA's ability to deal with nonstationary signals and to isolate significant signal components from noise, a task at which traditional spectral methods often fail.

The proposed framework should be interpreted as a transferable analytical protocol rather than a site-specific model. While the modeling parameters obtained in this study are specific to the Cartaro karst system, the methodological workflow can be applied to other karst aquifers provided that it is recalibrated. Moreover, the framework could be made adaptive through periodic updating of the calibration window and re-estimation of model parameters as new meteorological and discharge data become available using site-specific hydroclimatic and discharge data.

Finally, the approach presented here could be further developed into a graphical forecasting interface, however, such an implementation falls beyond the scope of the present study.

## Supporting information

S1 File Supporting information tables. (DOCX)

S2 File Supporting information Table 3. (CSV)

S1 Appendix Singular spectrum analysis (SSA). (DOCX)

## References

[1] Giorgi G, Ringwood JV. Comparing nonlinear hydrodynamic forces in heaving point absorbers and oscillating wave surge converters. J Ocean Eng Mar Energy. 2017;4(1):25–35. doi: 10.1007/s40722-017-0098-2

[2] Milly PCD, Dunne KA, Vecchia AV. Global pattern of trends in streamflow and water availability in a changing climate. Nature. 2005;438(7066):347–50. doi: 10.1038/nature04312 16292308

[3] Hirabayashi Y, Mahendran R, Koirala S, Konoshima L, Yamazaki D, Watanabe S, et al. Global flood risk under climate change. Nature Clim Change. 2013;3(9):816–21. doi: 10.1038/nclimate1911

[4] Chan D, Wu Q. Significant anthropogenic-induced changes of climate classes since 1950. Scientific Reports. 2015;5(1):13487. doi: 10.1038/srep13487 26316255 PMC4551970

[5] Turco M, Palazzi E, von Hardenberg J, Provenzale A. Observed climate change hotspots. Geophysical Research Letters. 2015;42(9):3521–8. doi: 10.1002/2015gl063891

[6] Baronetti A, Menichini M, Provenzale A. Vegetation response to droughts: The case of northern Italy. Intl Journal of Climatology. 2023;44(2):501–20. doi: 10.1002/joc.8340

[7] Llamas MR, Martínez-Santos P. Intensive groundwater use: a silent revolution that cannot be ignored. Water Sci Technol. 2005;51(8):167–74. doi: 10.2166/wst.2005.0254 16007945

[8] Taylor RG, Scanlon B, Döll P, Rodell M, van Beek R, Wada Y, et al. Ground water and climate change. Nature Clim Change. 2012;3(4):322–9. doi: 10.1038/nclimate1744

[9] Zhu Y, Balke K-D. Groundwater protection: what can we learn from Germany? J Zhejiang Univ Sci B. 2008;9(3):227–31. doi: 10.1631/jzus.B0710639 18357625 PMC2266878

[10] Zhang B, Meng F. Delineation methods and application of groundwater source protection zone. In: 2011 International Symposium on Water Resource and Environmental Protection, 2011. 66–9. 10.1109/iswrep.2011.5892945

[11] Doveri M, Menichini M, Cerrina Feroni A. Stable water isotope as fundamental tool in karst aquifer studies: some results from isotopic applications in the Apuan Alps carbonatic complexes (NW Tuscany). Italian Journal of Engineering Geology and Environment. 2013;2013(1):33–50.

[12] Doveri M, Menichini M, Scozzari A. Protection of groundwater resources: worldwide regulations and scientific approaches. Threats to the Quality of Groundwater Resources: Prevention and Control. Berlin, Heidelberg: Springer Berlin Heidelberg. 2015:13–30.

[13] Hiscock KM. Groundwater in the 21st century–meeting the challenges. Sustaining groundwater resources: A critical element in the global water crisis. Dordrecht: Springer Netherlands. 2011:207–25.

[14] Martínez Navarrete C, Grima Olmedo J, Durán Valsero JJ, Gómez Gómez JD, Luque Espinar JA, De la Orden Gomez JA. Groundwater protection in Mediterranean countries after the European water framework directive. Environmental Geology. 2008;54(3):537–49.

[15] Siebert S, Burke J, Faures JM, Frenken K, Hoogeveen J, Döll P, et al. Groundwater use for irrigation – a global inventory. Hydrol Earth Syst Sci. 2010;14(10):1863–80. doi: 10.5194/hess-14-1863-2010

[16] Ford D, Williams PD. Karst hydrogeology and geomorphology. John Wiley & Sons. 2007.

[17] Menichini M, Doveri M, Piccini L. Hydrogeological and geochemical overview of the karst aquifers in the Apuan Alps (Northwestern Tuscany, Italy). AS-ITJGW. 2016. doi: 10.7343/as-2016-198

[18] Maimone M. Defining and managing sustainable yield. Ground Water. 2004;42(6–7):809–14. doi: 10.1111/j.1745-6584.2004.tb02739.x 15584295

[19] Zhou Y. A critical review of groundwater budget myth, safe yield and sustainability. Journal of Hydrology. 2009;370(1–4):207–13. doi: 10.1016/j.jhydrol.2009.03.009

[20] Gleeson T, Wada Y, Bierkens MFP, van Beek LPH. Water balance of global aquifers revealed by groundwater footprint. Nature. 2012;488(7410):197–200. doi: 10.1038/nature11295 22874965

[21] Mangin A. Contribution à l’étude hydrodynamique des aquifères karstiques. Université de Dijon. 1975.

[22] Dreiss SJ. Linear unit-response functions as indicators of recharge areas for large karst springs. Journal of Hydrology. 1983;61(1–3):31–44. doi: 10.1016/0022-1694(83)90233-0

[23] Bakalowicz M. Karst groundwater: a challenge for new resources. Hydrogeol J. 2005;13(1):148–60. doi: 10.1007/s10040-004-0402-9

[24] Kovács A, Sauter M. Modelling karst hydrodynamics. Methods in karst hydrogeology. CRC Press. 2014:201–22.

[25] Ghasemizadeh R, Hellweger F, Butscher C, Padilla I, Vesper D, Field M, et al. Review: Groundwater flow and transport modeling of karst aquifers, with particular reference to the North Coast Limestone aquifer system of Puerto Rico. Hydrogeol J. 2012;20(8):1441–61. doi: 10.1007/s10040-012-0897-4 23645996 PMC3640320

[26] Stevanović Z. Global distribution and use of water from karst aquifers. SP. 2018;466(1):217–36. doi: 10.1144/sp466.17

[27] Stevanović Z. Karst waters in potable water supply: a global scale overview. Environ Earth Sci. 2019;78(23). doi: 10.1007/s12665-019-8670-9

[28] Doveri M, Piccini L, Menichini M. Hydrodynamic and geochemical features of metamorphic carbonate aquifers and implications for water management: The Apuan Alps (NW Tuscany, Italy) case study. Karst Water Environment: Advances in Research, Management and Policy. Cham: Springer International Publishing. 2018:209–49.

[29] Pulido-Bosch A. Principles of Karst Hydrogeology. Springer International Publishing. 2021. doi: 10.978

[30] Jourde H, Wang X. Advances, challenges and perspective in modelling the functioning of karst systems: a review. Environ Earth Sci. 2023;82(17). doi: 10.1007/s12665-023-11034-7

[31] Goldscheider N, Drew D. Methods in karst hydrogeology. Crc Press. 2014.

[32] Kuniansky EL, Bellino JC, Dixon JF. Transmissivity of the upper Floridan aquifer in Florida and parts of Georgia, South Carolina, and Alabama. US Geological Survey. 2012.

[33] Helsel DR, Hirsch RM. Statistical methods in water resources. Elsevier. 1993.

[34] Fiorillo F, Petitta M, Preziosi E, Rusi S, Esposito L, Tallini M. Long-term trend and fluctuations of karst spring discharge in a Mediterranean area (central-southern Italy). Environ Earth Sci. 2014;74(1):153–72. doi: 10.1007/s12665-014-3946-6

[35] Rahbar A, Mirarabi A, Nakhaei M, Talkhabi M, Jamali M. A Comparative Analysis of Data-Driven Models (SVR, ANFIS, and ANNs) for Daily Karst Spring Discharge Prediction. Water Resour Manage. 2022;36(2):589–609. doi: 10.1007/s11269-021-03041-9

[36] Duran L, Massei N, Lecoq N, Fournier M, Labat D. Analyzing multi-scale hydrodynamic processes in karst with a coupled conceptual modeling and signal decomposition approach. Journal of Hydrology. 2020;583:124625. doi: 10.1016/j.jhydrol.2020.124625

[37] Schuler P, Duran L, Johnston P, Gill L. Quantifying and Numerically Representing Recharge and Flow Components in a Karstified Carbonate Aquifer. Water Resour Res. 2020;56(11):e2020WR027717. doi: 10.1029/2020WR027717 33518822 PMC7816274

[38] Fleury P, Plagnes V, Bakalowicz M. Modelling of the functioning of karst aquifers with a reservoir model: Application to Fontaine de Vaucluse (South of France). Journal of Hydrology. 2007;345(1–2):38–49. doi: 10.1016/j.jhydrol.2007.07.014

[39] Kurtulus B, Razack M. Modeling daily discharge responses of a large karstic aquifer using soft computing methods: Artificial neural network and neuro-fuzzy. Journal of Hydrology. 2010;381(1–2):101–11. doi: 10.1016/j.jhydrol.2009.11.029

[40] Scozzari A, Brozzo G. Making use of continuous measurements for change detection purposes: an application to water distribution networks. In: 2017 IEEE International Instrumentation and Measurement Technology Conference (I2MTC), 2017:1–6. 10.1109/i2mtc.2017.7969738

[41] Hochreiter S, Schmidhuber J. Long short-term memory. Neural Comput. 1997;9(8):1735–80. doi: 10.1162/neco.1997.9.8.1735 9377276

[42] Hu YH, Hwang JN. Handbook of neural network signal processing. CRC Press. 2001.

[43] Xiong L, O’Connor KM. Comparison of four updating models for real-time river flow forecasting. Hydrological Sciences Journal. 2002;47(4):621–39. doi: 10.1080/02626660209492964

[44] Chang N-B, Wen CG, Chen YL. A fuzzy multi-objective programming approach for optimal management of the reservoir watershed. European Journal of Operational Research. 1997;99(2):289–302. doi: 10.1016/s0377-2217(96)00019-7

[45] Özelkan EC, Duckstein L. Fuzzy conceptual rainfall–runoff models. Journal of Hydrology. 2001;253(1–4):41–68. doi: 10.1016/s0022-1694(01)00430-9

[46] Yu P-S, Yang T-C. Using synthetic flow duration curves for rainfall-runoff model calibration at ungauged sites. Hydrol Process. 2000;14(1):117–33. doi: 10.1002/(sici)1099-1085(200001)14:1<117::aid-hyp914>3.0.co;2-q

[47] Lu R-S, Lo S-L. Diagnosing reservoir water quality using self-organizing maps and fuzzy theory. Water Res. 2002;36(9):2265–74. doi: 10.1016/s0043-1354(01)00449-3 12108719

[48] Bárdossy A. The use of fuzzy rules for the description of elements of the hydrological cycle. Ecological Modelling. 1996;85(1):59–65. doi: 10.1016/0304-3800(95)00011-9

[49] Schulz K, Huwe B. Water flow modeling in the unsaturated zone with imprecise parameters using a fuzzy approach. Journal of Hydrology. 1997;201(1–4):211–29. doi: 10.1016/s0022-1694(97)00038-3

[50] Kurtulus B, Razack M. Evaluation of the ability of an artificial neural network model to simulate the input-output responses of a large karstic aquifer: the La Rochefoucauld aquifer (Charente, France). Hydrogeol J. 2006;15(2):241–54. doi: 10.1007/s10040-006-0077-5

[51] Mangin A. Pour une meilleure connaissance des systèmes hydrologiques à partir des analyses corrélatoire et spectrale. Journal of Hydrology. 1984;67(1–4):25–43. doi: 10.1016/0022-1694(84)90230-0

[52] Larocque M, Mangin A, Razack M, Banton O. Contribution of correlation and spectral analyses to the regional study of a large karst aquifer (Charente, France). Journal of Hydrology. 1998;205(3–4):217–31. doi: 10.1016/s0022-1694(97)00155-8

[53] Mathevet T, Lepiller M l., Mangin A. Application of time-series analyses to the hydrological functioning of an Alpine karstic system: the case of Bange-L’Eau-Morte. Hydrol Earth Syst Sci. 2004;8(6):1051–64. doi: 10.5194/hess-8-1051-2004

[54] Padilla A, Pulido-Bosch A. Study of hydrographs of karstic aquifers by means of correlation and cross-spectral analysis. Journal of Hydrology. 1995;168(1–4):73–89. doi: 10.1016/0022-1694(94)02648-u

[55] Thomson DJ. Spectrum estimation and harmonic analysis. Proc IEEE. 1982;70(9):1055–96. doi: 10.1109/proc.1982.12433

[56] Thomson DJ. Quadratic-inverse spectrum estimates: applications to palaeoclimatology. Philosophical Transactions of the Royal Society of London Series A: Physical and Engineering Sciences. 1990;332(1627):539–97. doi: 10.1098/rsta.1990.0130

[57] Alessio SM. Digital signal processing and spectral analysis for scientists: concepts and applications. 2015.

[58] Paluš M, Novotná D. Quasi-biennial oscillations extracted from the monthly NAO index and temperature records are phase-synchronized. Nonlin Processes Geophys. 2006;13(3):287–96. doi: 10.5194/npg-13-287-2006

[59] Hasselmann K. Stochastic climate models part I. Theory. Tellus. 1976;28(6):473–85.

[60] Broomhead DS, King GP. Extracting qualitative dynamics from experimental data. Physica D: Nonlinear Phenomena. 1986;20(2–3):217–36. doi: 10.1016/0167-2789(86)90031-x

[61] Vautard R, Ghil M. Singular spectrum analysis in nonlinear dynamics, with applications to paleoclimatic time series. Physica D: Nonlinear Phenomena. 1989;35(3):395–424.

[62] Vautard R, Yiou P, Ghil M. Singular-spectrum analysis: A toolkit for short, noisy chaotic signals. Physica D: Nonlinear Phenomena. 1992;58(1–4):95–126. doi: 10.1016/0167-2789(92)90103-t

[63] Keppenne CL, Ghil M. Adaptive filtering and prediction of the Southern Oscillation index. J Geophys Res. 1992;97(D18):20449–54. doi: 10.1029/92jd02219

[64] Ghil M, Yiou P. Spectral Methods: What They Can and Cannot do for Climatic Time Series. Decadal Climate Variability. Springer Berlin Heidelberg. 1996:445–82. doi: 10.1007/978-3-662-03291-6_11

[65] Allen MR, Smith LA. Monte Carlo SSA: Detecting irregular oscillations in the Presence of Colored Noise. J Climate. 1996;9(12):3373–404. doi: 10.1175/1520-0442(1996)009<3373:mcsdio>2.0.co;2

[66] Mann ME, Lees JM. Robust estimation of background noise and signal detection in climatic time series. Climatic Change. 1996;33(3):409–45. doi: 10.1007/bf00142586

[67] Ghil M, Taricco C. Advanced spectral-analysis methods. Past and present variability of the solar-terrestrial system: measurement, data analysis and theoretical models. Ios Press. 1997:137–59.

[68] Golyandina N, Nekrutkin V, Zhigljavsky AA. Analysis of time series structure: SSA and related techniques. CRC press. 2001.

[69] Ghil M, Allen MR, Dettinger MD, Ide K, Kondrashov D, Mann ME, et al. Advanced spectral methods for climatic time series. Reviews of Geophysics. 2002;40(1). doi: 10.1029/2000rg000092

[70] Kondrashov D, Ghil M. Spatio-temporal filling of missing points in geophysical data sets. Nonlin Processes Geophys. 2006;13(2):151–9. doi: 10.5194/npg-13-151-2006

[71] Kondrashov D, Shprits Y, Ghil M. Gap filling of solar wind data by singular spectrum analysis. Geophysical Research Letters. 2010;37(15). doi: 10.1029/2010gl044138

[72] Golyandina N, Zhigljavsky A. SSA for forecasting, interpolation, filtration and estimation. Singular Spectrum Analysis for Time Series. Berlin, Heidelberg: Springer Berlin Heidelberg. 2013:71–119.

[73] Groth A, Ghil M. Monte Carlo Singular Spectrum Analysis (SSA) Revisited: Detecting Oscillator Clusters in Multivariate Datasets. Journal of Climate. 2015;28(19):7873–93. doi: 10.1175/jcli-d-15-0100.1

[74] Zhigljavsky A. Singular Spectrum Analysis for Time Series. School of Mathematics, Cardiff University. 2016.

[75] Chollet F. Deep learning with Python. Simon and Schuster. 2021.

[76] Marques CAF, Ferreira JA, Rocha A, Castanheira JM, Melo-Gonçalves P, Vaz N, et al. Singular spectrum analysis and forecasting of hydrological time series. Physics and Chemistry of the Earth, Parts A/B/C. 2006;31(18):1172–9. doi: 10.1016/j.pce.2006.02.061

[77] Giannecchini R. Relationship between rainfall and shallow landslides in the southern Apuan Alps (Italy). Nat Hazards Earth Syst Sci. 2006;6(3):357–64. doi: 10.5194/nhess-6-357-2006

[78] Civita M. Carta della vulnerabilità all’inquinamento degli acquiferi delle Alpi Apuane. Consiglio Nazionale delle Ricerche. 1991.

[79] Piccini L, Pranzini G, Tedici L, Forti P. Le risorse idriche dei complessi carbonatici del comprensorio apuo-versiliese. Quaderni Geologia Applicata. 1999;6(1):61–78.

[80] Menichini M, Da Prato S, Doveri M, Ellero A, Lelli M, Masetti G, et al. An integrated methodology to define Protection Zones for groundwaterbased drinking water sources: an example from the Tuscany Region, Italy. AS-ITJGW. 2015;4(1). doi: 10.7343/as-102-15-0129

[81] Drysdale R, Pierotti L, Piccini L, Baldacci F. Suspended sediments in karst spring waters near Massa (Tuscany), Italy. Environmental Geology. 2001;40(8):1037–50. doi: 10.1007/s002540100311

[82] Molli G, Doveri M, Manzella A, Bonini L, Botti F, Menichini M, et al. Surface-subsurface structural architecture and groundwater flowof the Equi Terme hydrothermal area, northern Tuscany Italy. IJG. 2015;134(3):442–57. doi: 10.3301/ijg.2014.25

[83] Thornton PE, Running SW, White MA. Generating surfaces of daily meteorological variables over large regions of complex terrain. Journal of Hydrology. 1997;190(3–4):214–51. doi: 10.1016/s0022-1694(96)03128-9

[84] Fisher RA. The distribution of the partial correlation coefficient. Metron. 1924;3:329–32.

[85] Fisher RA. Statistical methods for research workers. Breakthroughs in statistics: Methodology and distribution. New York, NY: Springer New York. 1970:66–70.

[86] Stuart A, Ord K, Arnold S. Kendall’s Advanced Theory of Statistics. 6th ed. Wiley. 2010.

[87] Akaike H. A new look at the statistical model identification. IEEE Trans Automat Contr. 1974;19(6):716–23. doi: 10.1109/tac.1974.1100705

[88] Box G, Jenkins GM. Analysis: Forecasting and Control. San Francisco. 1976.

[89] Childers DG. Modern spectrum analysis. 1978.

[90] Montgomery DC, Johnson LA, Gardiner JS. Forecasting and time series analysis. New York: McGraw-Hill. 1990.

[91] Penland C, Ghil M, Weickmann KM. Adaptive filtering and maximum entropy spectra with application to changes in atmospheric angular momentum. J Geophys Res. 1991;96(D12):22659–71. doi: 10.1029/91jd02107

[92] Alessio S, Vivaldo G, Taricco C, Ghil M. Natural variability and anthropogenic effects in a Central Mediterranean core. Clim Past. 2012;8(2):831–9. doi: 10.5194/cp-8-831-2012

[93] Goldscheider N, Drew D. Methods in karst hydrogeology. IAH: International Contributions to Hydrogeology, 26.

[94] Hartmann A, Goldscheider N, Wagener T, Lange J, Weiler M. Karst water resources in a changing world: Review of hydrological modeling approaches. Rev Geophys. 2014;52(3):218–42. doi: 10.1002/2013rg000443

[95] Giannecchini R. Rainfall triggering soil slips in the southern Apuan Alps (Tuscany, Italy). Adv Geosci. 2005;2:21–4. doi: 10.5194/adgeo-2-21-2005

[96] Natali S, Zanchetta G, Luppichini M, Doveri M, Isola I, Giannecchini R. Assessing moisture origin as a potential driver of event-based precipitation isotope variability in a Western Mediterranean catchment (Apuan Alps, Italy). Clim Dyn. 2025;63(3). doi: 10.1007/s00382-025-07650-7

[97] Fiorillo F, Doglioni A. The relation between karst spring discharge and rainfall by cross-correlation analysis (Campania, southern Italy). Hydrogeol J. 2010;18(8):1881–95. doi: 10.1007/s10040-010-0666-1

[98] Kutner MH, Nachtsheim CJ, Neter J, Li W. Applied linear statistical models. New York: McGraw, Irwin.

[99] Dormann CF, Elith J, Bacher S, Buchmann C, Carl G, Carré G, et al. Collinearity: a review of methods to deal with it and a simulation study evaluating their performance. Ecography. 2012;36(1):27–46. doi: 10.1111/j.1600-0587.2012.07348.x

[100] Graham MH. Confronting multicollinearity in ecological multiple regression. Ecology. 2003;84(11):2809–15. doi: 10.1890/02-3114

[101] Wunsch A, Liesch T, Cinkus G, Ravbar N, Chen Z, Mazzilli N, et al. Karst spring discharge modeling based on deep learning using spatially distributed input data. Hydrol Earth Syst Sci. 2022;26(9):2405–30. doi: 10.5194/hess-26-2405-2022

[102] Rudolph MG, Collenteur RA, Kavousi A, Giese M, Wöhling T, Birk S, et al. A data-driven approach for modelling Karst spring discharge using transfer function noise models. Environ Earth Sci. 2023;82(13):339. doi: 10.1007/s12665-023-11012-z 37366470 PMC10290613

[103] Dikici M, Burgan HI. Exploring the effect of meteorological and hydrological trends on groundwater drought index: The case of Seyhan Basin. Ecohydrology. 2025;18(6):e70110.

[104] Naveau P, Hannart A, Ribes A. Statistical Methods for Extreme Event Attribution in Climate Science. Annu Rev Stat Appl. 2020;7(1):89–110. doi: 10.1146/annurev-statistics-031219-041314

[105] Katz RW. Statistics of extremes in climate change. Climatic Change. 2010;100(1):71–6. doi: 10.1007/s10584-010-9834-5

