# Supplementary material for: Data-driven modeling of spring discharge in a dynamic karst aquifer using multivariate models and spectral analysis
Source: PLoS One. 2026 Aug 11;21(8):e0351623. doi: 10.1371/journal.pone.0351623 (PMC13460612; doi:10.1371/journal.pone.0351623)
Supplement: Supplementary file 1 [file pone.0351623.s001.docx]

**Supporting Information**

**Data-driven modeling of spring discharge in a dynamic karst aquifer using multivariate models and spectral analysis**

Gianna Vivaldo^1,2^, Brunella Raco^1*^, Matia Menichini^1^, Giulio Masetti^1^, Luca Fibbi^3,4^, Bernardo Gozzini^3,4^, Daniele Grifoni^3,4^, Marco Doveri^1,5^

^1^ Institute of Geoscience and Earth Resources, National Research Council, Pisa, Italy

^2^ National Biodiversity Future Centre, Palermo, Italy

^3^ Institute of Bioeconomy, National Research Council, Sesto Fiorentino (FI), Italy

^4^ LaMMA Consortium, Sesto Fiorentino (FI), Italy

^5^ Department of Earth Science, University of Pisa, Pisa, Italy

*Corresponding author: brunella.raco@cnr.it

**S1 Cross-correlation analysis**

Tables S1.1, S1.2, S1.3 and S1.4 show the Pearson correlation indices for several combinations of the regressors, for each class of model. Study period for Table S1.1, S1.2, S1.3: Jan 2016-Dec 2019. Study period for Table S1.4: Jan 2018-Dec 2019.

|  | ***P_m20*** | ***P_m45*** | ***P_m60*** | ***P_m90*** | ***P_m120*** |
| --- | --- | --- | --- | --- | --- |
| ***P*** | 0.18 | 0.14 | 0.11 | 0.1 | 0.1 |
| ***T*** | -0.48 | -0.5 | -0.5 | -0.48 | -0.36 |
| ***Q*** | 0.42 | 0.58 | 0.64 | 0.71 | 0.71 |
|  | ***T_m20*** | ***T_m45*** | ***T_m60*** | ***T_m90*** | ***T_m120*** |
| ***P*** | -0.18 | -0.14 | -0.12 | -0.088 | -0.088 |
| ***T*** | 0.9 | 0.83 | 0.77 | 0.61 | 0.61 |
| ***Q*** | -0.59 | -0.69 | -0.72 | -0.76 | -0.76 |

**Table S1.1**. Pearson correlation coefficients computed between averaged versions of raw data used as regressors of class 1 model. Rolling mean is computed over *m*-past values (days).

|  | ***P_L5*** | ***P_L15*** | ***P_L30*** | ***P_L60*** | | ***P_L90*** | ***P_m30_L5*** | ***P_m30_L15*** | ***P_m30_L30*** | ***P_m30_L60*** | ***P_m30_L90*** |
| --- | --- | --- | --- | --- | --- | --- | --- | --- | --- | --- | --- |
| ***P*** | 0.09 | 0.077 | 0.0066 | -0.0086 | | 0.054 | 0.11 | 0.072 | 0.011 | 0.00041 | 0.033 |
| ***T*** | -0.2 | -0.16 | -0.13 | -0.083 | | -0.0025 | -0.47 | -0.38 | -0.28 | -0.15 | 0.12 |
| ***Q*** | 0.13 | 0.21 | 0.21 | 0.14 | | 0.068 | 0.53 | 0.54 | 0.5 | 0.35 | 0.23 |
|  |  | | | | ***T_L30*** | | ***T_L60*** | ***T_L90*** | ***T_m30_L30*** | ***T_m30_L60*** | ***T_m30_L90*** |
| ***P*** |  | | | | -0.13 | | -0.022 | 0.034 | -0.071 | -0.0015 | 0.065 |
| ***T*** |  | | | | 0.7 | | 0.38 | -0.017 | 0.6 | 0.21 | -0.23 |
| ***Q*** |  | | | | -0.69 | | -0.67 | -0.48 | -0.74 | -0.64 | -0.38 |

**Table S1.2**. Pearson correlation coefficients computed between shifted and averaged versions of raw data used as regressors of class 2 model. Rolling mean is computed over *m*-past values (days). The time series shifts in days are indicated by the *L* parameter.

|  | ***Q_L1*** | ***Q_m5*** | ***Q_m8*** | ***Q_m10*** |
| --- | --- | --- | --- | --- |
| ***P*** | 0.083 | 0.075 | 0.062 | 0.057 |
| ***T*** | -0.45 | -0.45 | -0.44 | -0.43 |
| ***Q*** | 0.84 | 0.89 | 0.88 | 0.87 |

**Table S1.3**. Pearson correlation coefficients computed between shifted and averaged versions of the dependent variable, *Q*, used as “memory term” in class 3a model. Rolling mean is computed over *m*-past values (days). The time series shifts in days are indicated by the *L* parameter.

|  | ***P_m20*** | ***P_m45*** | ***P_m60*** | ***P_m90*** | ***P_m120*** |
| --- | --- | --- | --- | --- | --- |
| ***P*** | 0.18 | 0.16 | 0.14 | 0.098 | 0.071 |
| ***T*** | -0.46 | -0.5 | -0.49 | -0.39 | -0.26 |
| ***Q*** | 0.38 | 0.55 | 0.62 | 0.71 | 0.68 |
|  | ***T_m20*** | ***T_m45*** | ***T_m60*** | ***T_m90*** | ***T_m120*** |
| ***P*** | -0.16 | -0.14 | -0.12 | -0.035 | -0.035 |
| ***T*** | 0.91 | 0.83 | 0.76 | 0.56 | 0.56 |
| ***Q*** | -0.64 | -0.73 | -0.76 | -0.79 | -0.79 |
|  | ***Q_L1*** | ***Q_m5*** | ***Q_m8*** | ***Q_m10*** |  |
| ***P*** | 0.1 | 0.088 | 0.073 | 0.071 |  |
| ***T*** | -0.51 | -0.51 | -0.50 | -0.49 |  |
| ***Q*** | 0.94 | 0.93 | 0.92 | 0.91 |  |

**Table S1.4**. Pearson correlation coefficients computed between shifted and averaged versions of the dependent variable, *Q*, used as “memory term” in class 3b model. Rolling mean is computed over *m*-past values (days). The time series shifts in days are indicated by the *L* parameter.

**S2 Ordinary least square (OLS) regression statistics**

Table S2.1 shows the comparison of ordinary least square (OLS) regression statistics for each class of model.

Tables S2.2–S2.5 show the OLS regression parameters statistics for each class of model. For each model class, the following statistical parameters are reported: the coefficient term (*coef*), the standard error or standard deviation (*std.err*), the t-statistics (*t*), the p-value (*P>|t|*), and the confidence intervals at the *𝛼 = 0.05* significance level.

Study period for class 1, 2, 3a models: Jan 2016-Dec 2019. Study period for class 3b models: Jan 2018-Dec 2019.

| **MODEL CLASS** |  | | | | | |
| --- | --- | --- | --- | --- | --- | --- |
| **1** | *Q ~ P_m60 + T_m45 + T_m90* | | | | | |
|  | ***R^2^*** | ***Adj. R^2^*** | ***F-stat.*** | ***Log-lik.*** | ***AIC*** | ***BIC*** |
|  | 0.645 | 0.644 | 827.8 | -7182 | 1.437E+04 | 1.439E+04 |
| **2** | *Q ~ P_m30_L15 + T_m30_L30 + T_m30_L60* | | | | | |
|  | ***R^2^*** | ***Adj. R^2^*** | ***F-stat.*** | ***Log-lik.*** | ***AIC*** | ***BIC*** |
|  | 0.614 | 0.613 | 726.1 | -7243.9 | 1.450E+04 | 1.452E+04 |
| **3a** | *Q ~ Q_m5 + P_m20 + T_m60* | | | | | |
|  | ***R^2^*** | ***Adj. R^2^*** | ***F-stat.*** | ***Log-lik.*** | ***AIC*** | ***BIC*** |
|  | 0.798 | 0.797 | 1839 | -6943 | 1.389E+04 | 1.391E+04 |
| **3b** | *Q ~ Q_m5 + P_m45 + T_m90* | | | | | |
|  | ***R^2^*** | ***Adj. R^2^*** | ***F-stat.*** | ***Log-lik.*** | ***AIC*** | ***BIC*** |
|  | 0.880 | 0.879 | 1553 | -2925.7 | 5.859E+03 | 5.877E+03 |

**Table S2.1** Ordinary least square (OLS) regression results for each class of model. The model class is reported in the first column. The best fit is also indicated for each class of model. The following statistics are reported: coefficient of determination (*R^2^*), adjusted coefficient of determination (*Adj. R^2^*), F test (*F-stat.*), model log-likelihood (*Log-lik.*), Akaike information criterion (*AIC*), Bayesian information criterion (*BIC*).

| ***Class 1*** | ***coef*** | ***std. err*** | ***t*** | ***P>\|t\|*** | ***[0.025*** | ***0.975]*** |
| --- | --- | --- | --- | --- | --- | --- |
| *Intercept* | 313.4246 | 5.761 | 54.408 | 0.000 | 302.124 | 324.725 |
| *P_m60* | 9.5695 | 0.564 | 16.969 | 0.000 | 8.463 | 10.676 |
| *T_m45* | 3.1972 | 0.540 | 5.925 | 0.000 | 2.139 | 4.256 |
| *T_m90* | -10.9439 | 0.546 | -20.045 | 0.000 | -12.015 | -9.873 |

**Table S2.2**. Class 1 model: Parameters statistics from OLS regression.

| ***Class 2*** | ***coef*** | ***std. err*** | ***t*** | ***P>\|t\|*** | ***[0.025*** | ***0.975]*** |
| --- | --- | --- | --- | --- | --- | --- |
| *Intercept* | 336.0153 | 4.504 | 74.609 | 0.000 | 327.180 | 344.850 |
| *P_m30_L15* | 5.8205 | 0.417 | 13.950 | 0.000 | 5.002 | 6.639 |
| *T_m30_L30* | - 4.9662 | 0.429 | -11.564 | 0.000 | -5.809 | -4.124 |
| *T_m30_L60* | -3.2345 | 0.379 | -8.525 | 0.000 | -3.979 | -2.49 |

**Table S2.3**. Class 2 model: Parameters statistics from OLS regression.

| ***Class 3a*** | ***coef*** | ***std. err*** | ***t*** | ***P>\|t\|*** | ***[0.025*** | ***0.975]*** |
| --- | --- | --- | --- | --- | --- | --- |
| *Intercept* | 50.7235 | 7.840 | 6.470 | 0.000 | 35.345 | 66.102 |
| *Q_m5* | 0.8488 | 0.020 | 42.506 | 0.000 | 0.810 | 0.888 |
| *P_m20* | 0.9869 | 0.236 | 4.186 | 0.000 | 0.524 | 1.449 |
| *T_m60* | -1.2807 | 0.248 | -5.166 | 0.000 | -1.767 | -0.794 |

**Table S2.4**. Class 3a model: Parameters statistics from OLS regression.

| ***Class 3b*** | ***coef*** | ***std. err*** | ***t*** | ***P>\|t\|*** | ***[0.025*** | ***0.975]*** |
| --- | --- | --- | --- | --- | --- | --- |
| *Intercept* | 44.6671 | 9.782 | 4.566 | 0.000 | 25.458 | 63.877 |
| *Q_m5* | 0.8596 | 0.026 | 32.949 | 0.000 | 0.808 | 0.911 |
| *P_m45* | 1.0147 | 0.315 | 3.225 | 0.001 | 0.397 | 1.632 |
| *T_m90* | -1.0292 | 0.292 | -3.523 | 0.000 | -1.603 | -0.456 |

**Table S2.5**. Class 3b model: Parameters statistics from OLS regression.
