# Supplementary material for: Data-driven modeling of spring discharge in a dynamic karst aquifer using multivariate models and spectral analysis
Source: PLoS One. 2026 Aug 11;21(8):e0351623. doi: 10.1371/journal.pone.0351623 (PMC13460612; doi:10.1371/journal.pone.0351623)
Supplement: Supplementary file 2 [file pone.0351623.s003.docx]

**Appendix**

**Singular spectrum analysis (SSA)**

SSA is an advanced spectral analysis technique designed to get information about non-linear systems from short and noisy time series without appealing to the process governing equations. equations [60–74]. The method is nonparametric and provides data-adaptive filters which allow the decomposition of time series into statistically independent components or reconstructed components (*RCs*), classified as trends, harmonic and anharmonic oscillatory patterns and random noise. We refer to Ghil et al., 2022 [69] for details about the theoretical framework. In this work, Monte Carlo SSA (MC-SSA) method was used to perform signal-to-noise (S/N) separation against a red-noise null hypothesis [65, 73]. MC-SSA allows to overcome the limitation of the traditional denoising approach (i.e., the identification of a noise floor in a sequence of eigenvalues given in descending order) which becomes problematic when either the S/N ratio is not sufficiently large or the noise affecting the data is not simply white but “coloured” [57, 59]. Although SSA accepts white and locally white background noise to filter the signal, in our study red noise was chosen following Hasselman’s theory (1976) [59], according to which natural systems (including hydrological systems and, more generally, climatic systems) are stochastic processes in which long-term variability is amplified, leading to more energy (power) at lower frequencies.

SSA was combined with other spectral analysis tools – the maximum entropy method (MEM) and multi-taper method (MTM) [67] – for refining the interpretation of oscillatory behavior, since only the simultaneous and flexible application of more than one spectral method can assure reliable information on a given time series, especially when its signal-to-noise ratio is low [57, 67, 69], such as in our case. In the paper, we show only those oscillatory patterns significant to all methods.

The gap filling version of SSA [70-71] was applied to process datasets with uneven sampling due to missing observations. Gaps are filled in by using the temporal correlations of one-dimensional time series, thus accounting for the true signal embedded into the analyzed time series, only. With respect to traditional methods (e.g., linear fit and moving average), this approach respects the whole process temporal variability and not only the local one (i.e., near the gap). Gap filling can be tuned by changing the dimension of the SSA window length (*M*) and the number of eigenvalues or components to include in gap filling calculation (*C*). These parameters are chosen to cover the largest temporal correlations but, at the same time, to retain as much signal as possible without introducing random noise into the signal gap-filled reconstruction.

**SSA-AR prediction**

Single-channel time series prediction was based on the combination of and singular spectrum analysis (SSA) and classical low-order autoregressive (AR) models [63] as an innovative noise reduction strategy.

The basic idea is that the reconstructed components (RCs) in SSA are data-adaptive filtered signals, each of which is dominated by a narrow spectral peak. Such narrow-band signals allow much more robust predictions than broad-band ones [57-58, 63, 69], reducing noise propagation. All the individual predictions of each RC can then be linearly combined to form a partial reconstruction of the entire time series' future evolution [63, 69]. The SSA-AR method turns out to be most reliable and robust when the order of the autoregressive model fitted to the data (*M_AR_*) is of the same magnitude order of the SSA embedding dimension (or SSA filtering window, *M_SSA_*). This result follows from the SSA-AR algorithm definition, in particular the way the SSA lag-covariance matrix and Burg’s algorithm AR coefficients are computed. Moreover, both *M_AR_* and *M_SSA_* have not to be too large with respect to the length *N* of the time series, since the variance of the AR coefficients estimate increases with the order *M_AR_*.

In this work, prediction is performed with a maximum lead (*L_max_*) of one year. The prediction skill is evaluated through an iterative one-step-into-the-future forecasting procedure by cross validation over a test section (*N_test_*), following the approach by Alessio et al. (2012) [57]. More in details, according to the SSA-AR theory, given a *N*-length SSA reconstructed component (RC) and a maximum required lead *L_max_*, the test section length is defined as *N_test_ = N - 3M_SSA_ - L_max_*, and the lower and upper limits of the test section are defined as *N_test,in_ = 3M_SSA_*  and *N_test,fin_ = N - L_max_* , respectively. A fixed *K*-length sliding window will roll iteratively over the test section to provide cross-validation. The length of the sliding window is defined as *K* = *N_test_ - L_max_*, although it is recommended to check if *K* allows the lower frequency periodicity detected in the input series to be reproduced a significant number of times [69]. For a value *L* of the lead, the initial and final points of the sliding window are *x_in_ = 3M_SSA_ + L* and *x_fin_ = 3M_SSA_ + L + K*, respectively. Inside the test section, the fixed *K*-length window shifts *L* steps ahead, with *L* belonging to the range{*L*: 1≤ *L* ≤ *L_max_*}. For any position of the sliding window, corresponding to each lead time *L*, *K*-length pairs of predicted−true values are provided for computing the root-mean-square (RMS) forecast errors $R(L)$. Forecasting parameters are then optimized based on cross-validation results. The total prediction uncertainty is computed as the square root of the sum of the squared RMS errors of the individual RC forecast. In agreement with Vautard et al. (1992) [58], the learning section length is defined as *N_learn_ = N – N_test_ - L_max_* = *3M_SSA_*, with lower and upper boundaries defined as *N_learn,in_ = 1*  and *N_learn,fin_ =3M_SSA_*.
